# Antifungal Effect of Poly(methyl methacrylate) Coated with Polyelectrolyte Multilayers

**DOI:** 10.1021/acsomega.5c00883

**Published:** 2025-05-07

**Authors:** Klemen Bohinc, Anamarija Zore, Tina Velikonja, Franc Rojko, Roman Štukelj, Aleksander Učakar, Anže Abram, Nives Matijaković Mlinarić, Miha Čekada, Juraj Nikolić, Davor Kovačević

**Affiliations:** † Faculty of Health Sciences, 37663 University of Ljubljana, 1000 Ljubljana, Slovenia; ‡ 61790 Jožef Stefan Institute, Jamova cesta 39, 1000 Ljubljana, Slovenia; § Department of Chemistry, Faculty of Science, 117036 University of Zagreb, 10000 Zagreb, Croatia

## Abstract

Due to teeth loss, a large proportion of the elderly rely on full or partial dentures for esthetic, speaking, and eating reasons. A variety of polymers are used in the production of removable prostheses, with poly­(methyl methacrylate) (PMMA) being a widely used material for making denture bases. However, underdenture stomatitis caused by the fungi Candida albicans is still an open problem. The purpose of this work was to consider the impact of polyelectrolyte multilayer (PEM) coating on PMMA surfaces and the effect of the addition of sucrose on the adhesion properties of C. albicans. Two polyelectrolytes were applied for the formation of the PEM coating: poly­(allylamine) hydrochloride (PAH) and poly­(acrylic) acid (PAA). The uncoated and coated surfaces were characterized in terms of topography, surface potential, and hydrophobicity. The extent of adhesion of C. albicans to the surfaces was assessed by scanning electron microscopy. Results show that surfaces coated with negatively charged PAA as the PEM terminating layer adhere less C. albicans than uncoated PMMA or surfaces coated with positively charged PAH as the PEM terminating layer. The addition of sucrose increases the fungal adhesion extent of C. albicans to both types of coated surfaces, lowering the PAA antiadhesion properties. With the addition of sucrose, we were trying to mimic the impact of dentures on patients with a sugar-rich diet.

## Graphical abstract

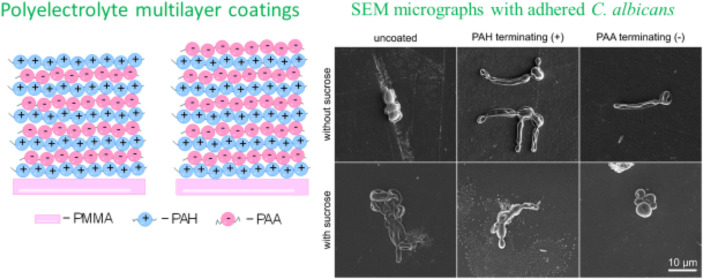

## 1 Introduction

An increasing number of partially or totally toothless patients has been reported globally.^1,2^ This is the consequence of the increasing aging of the general population. Available data show that the world’s population older than 60 will increase from 12 to 22% between 2015 and 2050.^3^ The lower-income population has only the possibility of obtaining total or partial dentures. On the other hand, a wealthy part of the population can ensure implant prosthetics. The total denture type of prosthetic reconstruction includes gingival-supported restoration with a large area of support on the soft tissues of the oral cavity. The most common denture base material is poly­(methyl methacrylate) (PMMA).^4,5^

PMMA has good physical and mechanical material properties: a high degree of biocompatibility, simple prosthesis production technology, low weight, low density, ease of finishing and polishing, stability in the oral environment, satisfactory esthetics, color matching ability, and reparability.^4–9^ Despite PMMA having been prepared for many years, it has some limitations such as limited mechanical resistance against impact, fatigue, and flexural strengths, thermal conductivity, solubility, water sorption, insufficient surface hardness, and fouling of microorganisms.^10,11^ Denture wearers are susceptible to prosthetic stomatitis, which causes chronic inflammation of the soft tissue under the denture and occurs in 67% of patients wearing dentures.^12^ *Candida* species, such as Candida albicans, are the most common microbes that cause denture stomatitis and have been on the World Health Organization fungal priority pathogens list as a critical priority group as a high-risk fungal pathogen.^13^

Various dental surface properties, including the PMMA surface, could be additionally enhanced by surface modifications. For that purpose, different coatings could be applied to improve the PMMA properties. Among various possible coatings, the formation of polyelectrolyte multilayers (PEMs) has already proved to be promising.^14^ This technique is adaptable to dental surfaces, as well as to other types of surfaces, as shown in our previous studies.^15–17^ PEMs obtained by alternate adsorption of oppositely charged polyelectrolytes (polycations and polyanions) are usually only a few nanometers thick. The benefit of such coatings is that they are robust and can be used for long-lasting surface modification. The large range of available polyelectrolytes can enable the preparation of coatings with adjustable biocompatible properties, as shown for β-peptides applied by Palecek et al. for the design of surfaces with antifungal activity against C. albicans.^18^

Various other types of coatings have been used to limit C. albicans adhesion to PMMA. Reduction of C. albicans was noticed after O_2_ plasma treatment, which caused increased surface free energy and decreased PMMA contact angles.^19^ The application of trimethylsilane plasma coating increased PMMA surface hydrophobicity and inhibited the adhesion of C. albicans.^20^ C. albicans growth inhibition was also achieved by PMMA coating with a silica-based coating containing hinokitiol.^21^ Furthermore, photopolymerized coatings of poly­(acrylic acid) and poly­(itaconic acid) on PMMA dentures effectively reduced the C. albicans growth and adhesion.^22^ Also, plant molecules such as Cnidium officinale extracts coated on the PMMA surface demonstrated antifungal properties against C. albicans.^23^ The multilayer coating made from positive amphiphilic quaternary ammonium chitosans and negative sodium alginate on PMMA effectively prevented initial fungal adhesion and biofilm formation.^24^ The *Candida* cells were free in the solution and avoided the multilayer coatings, especially positively charged chitosan, which demonstrated fungal-repelling effects.^24,25^ Multilayer coating of polypeptide histatin 5 (cationic) and hyaluronic acid (anionic) demonstrated that the outermost layer histatin 5 inhibited *Candida* adhesion and reduced biofilm formation.^26^ On the other hand, some coatings are not effective for inhibiting C. albicans biofilm formation; such results were obtained on PMMA surfaces coated with Parylene-C and demonstrated that Parylene-C does not prevent biofilm formation.^27^

When studying the antifungal effect of various surfaces, properties crucial for adhesion, such as roughness, wettability, surface energy, and ζ-potential (charge), should be examined. These properties are accessible by techniques such as scanning electron microscopy (SEM), profilometry, atomic force microscopy (AFM), tensiometry, and electrophoresis. The fungal coverage of PMMA surfaces coated with PEMs can be studied with SEM whose micrographs need to be analyzed by a preprocessing technique.

The aim of the study was to evaluate the influence of the PEM-coated PMMA surface on *Candida albicans* adhesion. As oppositely charged polyelectrolytes we used poly­(allylamine) hydrochloride (PAH) and poly­(acrylic) acid (PAA), which are synthetic weakly charged polyelectrolytes and have been widely used in the process of polyelectrolyte multilayer formation.^28–30^ We prepared polyelectrolyte multilayers consisting of nine layers (terminating layer PAH) and ten layers (terminating layer PAA) to evaluate the influence of such coatings on the antifungal properties of dental material. Additionally, the aim of our study was also to find out if the addition of sucrose would increase the extent of fungal adhesion of C. albicans to all types of studied surfaces.

## 2 Materials and Methods

### 2.1 PMMA Preparation

PMMA plates were prepared from the material Policold (producer Polident d.o.o, Volja Draga 42, SI-5293 Volja Draga, Slovenia), which is a cold polymerizing acrylate used to produce prostheses, repairs, and lining of prostheses by the casting technique. First, three-dimensional (3D) samples were printed from the plastic mass and duplicated with duplicating silicone. For each sample, 1.6 g of polymers (powder) and 1 g of monomers (liquid) were mixed. The mixture was poured into a mold and polymerized. The sample was polymerized for 20 min at temperatures between 40 and 45 °C and under a pressure of 3 bar. After the polymerization, all samples were sandblasted in a sandblaster with aluminum oxide (Al_2_O_3_) of size 50 μm. Also polished PMMA samples were prepared. The polishing machine with a pumice stone, deerskin, and polishing paste was used. Both sample types were thoroughly steam cleaned at the end thoroughly steam cleaned. PEM-coated samples were prepared for both sandblast and polished samples. Surfaces were cleaned with steam, labeled, and finally coated with PAH/PAA polyelectrolyte multilayers.

### 2.2 Polyelectrolyte Multilayer Preparation

Polyelectrolyte multilayers were prepared by alternated adsorption of poly­(allylamine) hydrochloride (Aldrich, *M*_w_ = 50,000) and poly­(acrylic acid) (Fluka, *M_n_* = 130,000) using standard layer-by-layer deposition method on polished and sandblasted PMMA surfaces (Scheme 1) according to the procedure suggested by Decher et al.^31^ Polyelectrolyte solutions were prepared in 3-morpholinopropane-1-sulfonic acid (MOPS) buffer (*c* = 0.01 mol dm^–3^). The concentration of both polyelectrolytes in their respective MOPS buffer solutions was 0.01 mol dm^–3^ (calculated by using the molar mass of the monomer unit). The pH of the solutions was adjusted to 7 using NaOH (*c* = 1 mol dm^–3^). Deposition of the multilayers was done using the following procedure: the substrate was dipped for 5 min in the respective polyelectrolyte solution, rinsed in deionized water for a total of 3 min (1 min in three separate glasses), and afterward, dried with argon gas (5.0 purity). The procedure was repeated until the desired number of layers were deposited on the substrate. Two types of multilayers were studied: PEMs containing 9 layers and having PAH as the terminating layer will be later denoted as PMMA–(PAH/PAA)_4_–PAH and PEMs containing 10 layers (PAA as terminating layer) as PMMA–(PAH/PAA)_5_.

**Scheme 1:**
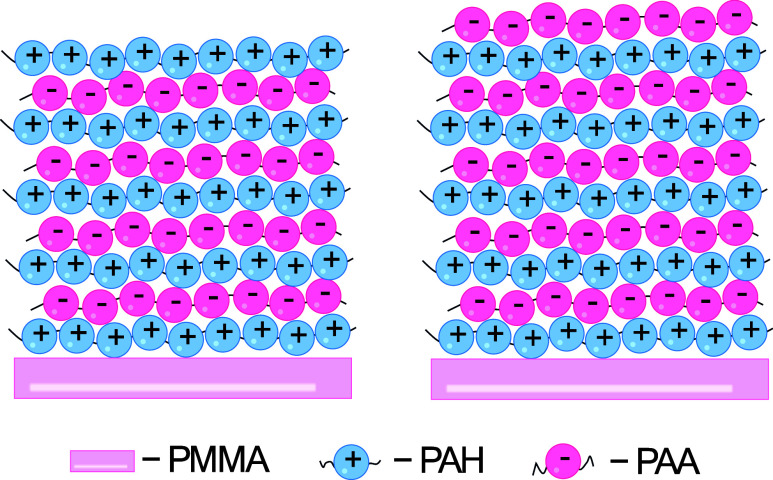
Polyelectrolyte Multilayer Coating Prepared on Poly­(methyl) Methacrylate Surface (PMMA) with Two Terminating Types of Layers, PAA (Pink Spheres) and PAH (Blue Spheres)^a^

a The coating procedure was conducted by alternating PAH/PAA layers. PAH—poly­(allylamine hydrochloride), PAA—poly­(acrylic) acid.

### 2.3 Profilometry Measurements

The roughness of PMMA samples was measured by a Taylor-Hobson Talsurf profilometer using a diamond tip with a radius of 2 μm. Line profiles were acquired with a total length of 4 mm. The mean roughness *R*_a_ was evaluated according to the ISO 4287 standard, using the Gaussian filter with a 0.8 mm cutoff. For each sample, we acquired three line profiles.^32^

### 2.4 Contact Angle Measurements

The static contact angle measurements were made with an Attension Theta T200-Basic Plus (Biolin Scientific) tensiometer. The droplet was placed on a sample by moving the tip vertically until contact between the water drop and the sample was made. After placing the droplet on the sample, images of the droplet (1216 px × 800 px) were taken for 8 s with a frequency of 5 fps through a CCD camera. Images were stored on a computer and the contour of the droplet on the solid surface was processed by the Young–Laplace equation using captured images.^33^ For each image, the contact angle on the left and right sides of the droplet was determined, and the average value of the contact angle was calculated. Five separate locations on the samples were measured to ensure a representative contact angle value.

### 2.5 ζ-Potential Measurements

The ζ-potential of uncoated and PEM-coated PMMA surfaces was measured with the electrokinetic analyzer (SurPASS 2, Anton Paar GmbH, Graz, Austria). At room conditions, the streaming potential has been measured within the capillary tube between 10 mm × 10 mm samples in 1 mM KCl solution at neutral pH.

### 2.6 AFM Measurements

Surface morphology and topography of PMMA substrates and the corresponding multilayers were determined by atomic force microscopy (AFM) using a Multimode 8E AFM apparatus from Bruker. NCHV-A silicon probes (Bruker) were 33 μm in width and 117 μm in length, with a resonance frequency of approximately 320 kHz. A nominal spring constant was 40 N/m. The tip height was 10–15 μm with a nominal radius of curvature of 8 nm. All AFM experiments were performed under ambient room conditions. AFM scans were performed on a 5 μm × 5 μm area with a scanning rate of 1 Hz and a picture resolution of 512 × 512 pixels. Afterward, the data was processed in NanoScope Scan 9.7. Images were corrected for bow and tilt by second-order flattening and were analyzed in NanoScope Analysis 2.0 software.

After 45 h of incubation, the PMMA samples with attached yeast were rinsed three times in phosphate-buffered saline (PBS) buffer and then fixed by using hot air. Subsequently, the samples were rinsed with distilled water and fixed again with hot air. The surfaces with attached yeasts were observed using a scanning electron microscope (SEM, Quanta 650, Thermo Fisher Scientific, Waltham, Massachusetts). Prior to imaging, the samples were coated with a thin layer of gold (approximately 5 nm thickness) using a gold coating device (BAL-TEC SCD 005, Baltec AG, Pfäffikon, Switzerland) under vacuum conditions of 5 × 10^–2^ Pa. The SEM imaging was conducted at a working distance of 10 mm, an accelerating voltage of 10 kV, and a vacuum level of 2 × 10^–4^ Pa by using the secondary electron sensor. Imaging has been conducted on several randomly selected areas on the sample surface to avoid observational bias.

The determination of the fungal coverage on SEM micrographs was manually outlined, and the images were converted to the binary form. The ImageJ software package (Version 1.50b, 2015, Wayne Rasband, National Institutes of Health, Bethesda, MD) (Schneider 2012) was used for this analysis.

### 2.7 Funghi/Yeast and Cell Adhesion Measurements

Candida albicans, like other microorganisms in the mouth microbiota, is typically present in small amounts on the oral mucosa. In individuals with a healthy immune system and good oral hygiene, this usually does not cause any problems. However, in people with weakened immune systems, like elderly people, and poor hygiene, *Candida* can proliferate and lead to inflammation (stomatitis). Treatment can often be time-consuming, so it is better to focus on prevention such as using a polyelectrolyte multilayer system.

In this study, *Candida* was cultured on YGC (Chloramphenicol Yeast Glucose) agar plates (Merck, Darmstadt, Germany). The overnight culture was then grown in Sabouraud broth (Bilife, Milano, Italy) at pH = 5.6 for 18 h at 37 °C and diluted 1:300 with fresh broth for further incubation at 37 °C for 45 h with samples to investigate biofilm formation. Incubations were performed without sucrose and in the presence of sucrose (2.5% concentration).

## 3 Results

### 3.1 PMMA Surface Roughness

The results of the profilometry measurements are presented in Table 1. There is a clear distinction between the polished samples (the grand average of all measurements is *R*_a_ = 0.08 μm) and the sandblasted samples (2.85 μm). The sandblasted samples demonstrated a 35 times rougher surface than the polished ones.

**Table 1:** Surface Roughness, *R*_a_, Data of Polished and Sandblasted PMMA Samples Measured by Profilometry

PMMA sample		*R*_a_/μm
polished	1	0.097 ± 0.02
	2	0.063 ± 0.01
	3	0.077 ± 0.01
sandblasted	1	1.93 ± 0.2
	2	3.64 ± 0.5
	3	2.21 ± 0.3

### 3.2 Contact Angle Measurements

Contact angle measurements were taken for two reasons. The first reason was to determine the wettability of both types of substrates, polished and sandblasted PMMA, and of PEMs with different terminating layers, 9th layer (PAH) and 10th layer (PAA), which were subjected to cell adhesion experiments. The second reason was to confirm the PEM film growth, as it was expected that PAH- and PAA-terminating layers would have different contact angles (with a zigzag pattern). The results of these experiments are presented in Figure 1.

**Figure 1:**
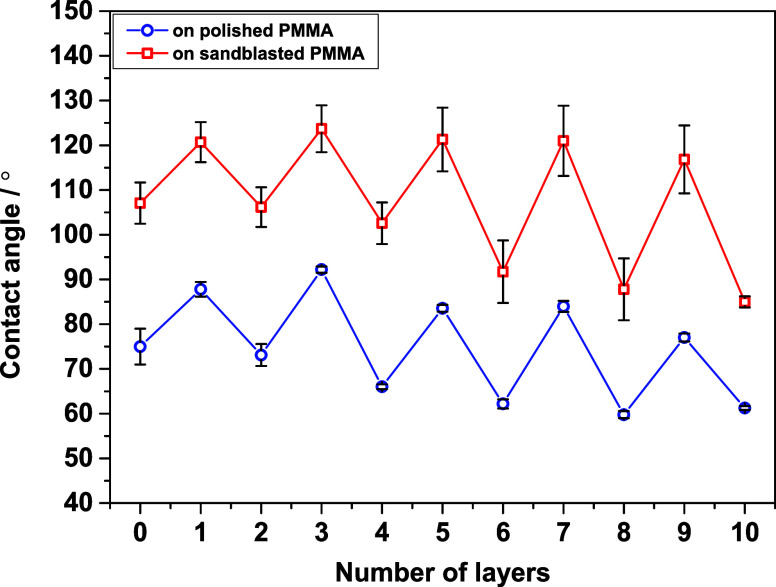
Contact angles obtained for every layer during the PAH/PAA multilayer buildup. The layer marked as “0” corresponds to the bare PMMA substrate. PAH is the terminating layer of odd-numbered layers, while the terminating PAA layer is denoted by even numbers.

As can be seen in Figure 1, contact angle measurements during the multilayer buildup follow a zigzag pattern with all measurements done on sandblasted PMMA having higher values of contact angles, indicating sandblasted PMMA films are hydrophobic. Looking at the individual values, the starting values for the PMMA substrate are (74.9 ± 4.0)° (polished) and (107.1 ± 4.6)° (sandblasted). The adsorption of the first and the second PAH layer makes the surface more hydrophobic, which is indicated by the rise in contact angle values by 10–20°. On the other hand, adsorption of every PAA layer decreases the contact angle, indicating that PAA makes the surface more hydrophilic. As mentioned before, cell adhesion experiments were done on PMMA substrates with nine (terminating layer PAH) and ten layers (terminating layer PAA). Contact angles of these layers are (77.0 ± 0.9)° and (116.8 ± 7.6)° for the 9th PAH layer, while (61.3 ± 0.4)° and (84.9 ± 1.2)° for the 10th PAA layer. As mentioned previously, the lower contact angle corresponds to more hydrophilic PEMs fabricated on polished PMMA substrate, while the higher contact angles correspond to more hydrophobic PEMs built up on sandblasted PMMA.

These results of zigzag pattern contact angle measurements during buildup of PAH/PAA multilayers were also reported on a different substrate (silica wafer)^15^ and is a typical behavior if the two polyelectrolytes which are used to fabricate the PEM have different wetting properties.

### 3.3 Surface Morphology and Topography

As mentioned before, AFM was used to investigate the surface morphology and topography of prepared PMMA surfaces and their corresponding PEMs. Polished PMMA substrate and its multilayers could be easily analyzed by AFM, but unfortunately, the surface of sandblasted PMMA proved to be too rough for AFM experiments and could not be investigated by this method. Representative AFM images of polished PMMA substrate without the multilayer and representative pictures of the 9th PAH layer and 10th PAA layer are shown in Figure 2.

**Figure 2:**
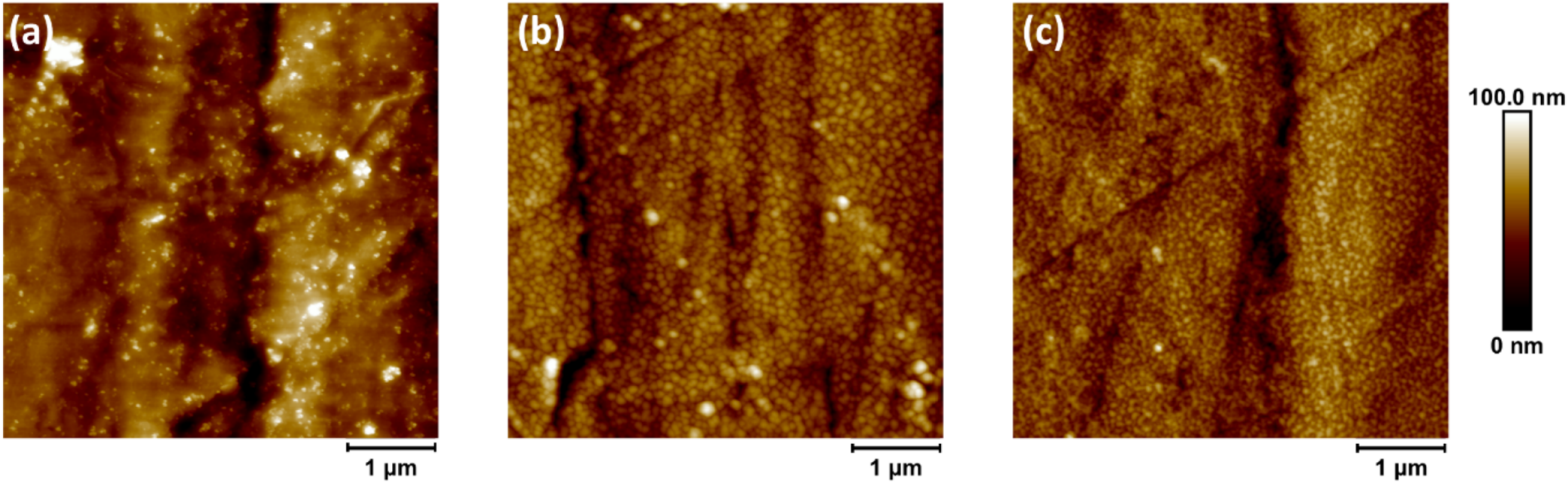
AFM images of the (a) polished PMMA surface; (b) polished PMMA-(PAH/PAA)_4_-PAH multilayer; and (c) polished PMMA-(PAH/PAA)_5_ multilayer.

Immediately upon comparison of Figure 2(a–c), the obvious differences can be observed. In Figure 2(a), the PMMA surface is smooth with irregular bumps on it, most likely a residue of the polishing material. In addition, deep straight rifts can be seen throughout the surface, which correspond to markings of the used polishing equipment. On the other hand, differences between Figure 2(b,c) are less noticeable but clear when compared to Figure 2(a). The surface in Figure 2(b,c) is fully covered by small grain-like structures. It should be noted that the grains have a more defined border and are larger when PAH is the terminating layer (Figure 2(b)) compared to the PAA-terminating layer (Figure 2(c)) where the grains seem smaller, more condensed, and seem to start to link to each other. Either way, these images confirm the successful adsorption of PAH/PAA multilayer to the PMMA surface.

### 3.4 ζ-Potential Measurements

To confirm the surface charge of PEM layers, as mentioned in the Experimental Section, ζ-potential measurements were performed in the neutral pH range. The results are listed in Table 2.

**Table 2:** ζ-Potential of the Uncoated Polished PMMA Surface and Polished PMMA Surface Coated with PEMs: PAH and PAA as Terminating Layers

sample	pH	ζ/mV
uncoated PMMA	6.72 ± 0.01	–56.3 ± 0.8
PMMA–(PAH/PAA)_4_–PAH	6.70 ± 0.01	51.7 ± 0.5
PMMA–(PAH/PAA)_5_	6.34 ± 0.01	–22.2 ± 0.4

As can be seen in Table 2, the ζ-potential of PAH- and PAA-terminating multilayers differs, as expected. Uncoated PMMA and PAA-terminating multilayers are negatively charged, whereas the PAH-terminating multilayer is positively charged. These results are in accordance with the charge properties of the PAH/PAA multilayers.

### 3.5 Cell Adhesion Measurements

Polished surfaces show larger C. albicans adhesion compared to sandblasted surfaces (Figure 3). On average, 95 C. albicans cells are adhered to the surface area of 5000 μm^2^ of sandblasted surfaces, whereas 437 cells are adhered to 5000 μm^2^ of polished PMMA surfaces. In addition, prolonged hypha structures were observed on sandblasted surfaces. Sandblasted surfaces have complicated surface structures with a relatively high surface roughness. These types of surfaces show properties that are unfavorable for C. albicans adhesion. The presence of aluminum oxide in sand of sandblasted surface causes the morphological change in the pseudohyphae of C. albicans cells.^34^

**Figure 3:**
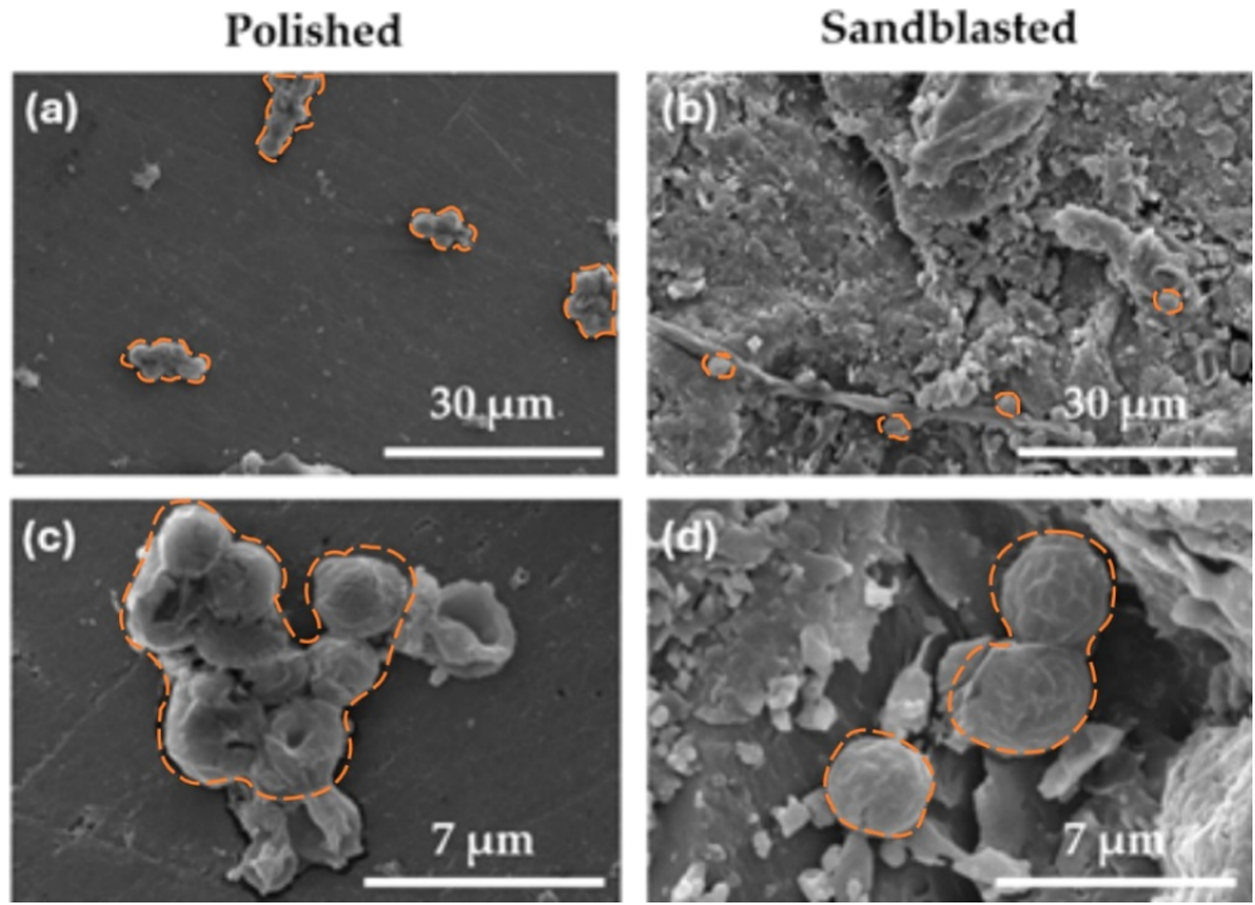
SEM images of (a, c) polished and (b, d) sandblasted bare (uncoated) PMMA surfaces with adhered C. albicans.

Table 3 summarizes the results for C. albicans adhesion on uncoated and coated PMMA surfaces without and with sucrose. Without sucrose, the lowest adhesion was observed on the PMMA surface coated with the terminating PAA layer. On the other hand, with sucrose, the lowest adhesion extent is obtained for uncoated PMMA surfaces and PMMA surface coated with PAA-terminating layer. SEM micrographs of polished uncoated and coated PMMA surfaces with adhered C. albicans are shown in Figure 4.

**Table 3:** Surface Coverage by C. albicans of Uncoated Polished PMMA and Polished PMMA Coated with PEMs Having Two Different Terminating Layers, PAH and PAA, as Determined by SEM. The number of cells per 5000 μm^2^ is given

sample	without sucrose	with sucrose
uncoated PMMA	52 ± 7	39 ± 3
PMMA–(PAH/PAA)_4_–PAH	55 ± 2	104 ± 5
PMMA–(PAH/PAA)_5_	21 ± 2	42 ± 2

**Figure 4:**
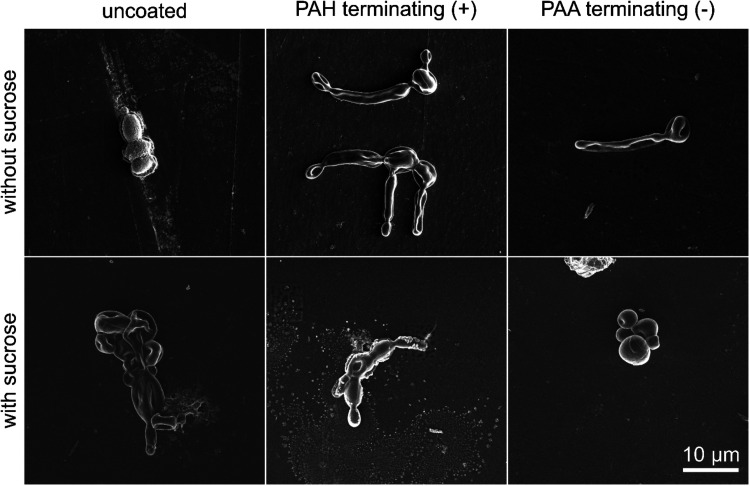
SEM micrographs of uncoated and coated polished PMMA surface with adhered C. albicans; first row: PMMA surface without a multilayer; second row: PEM terminating with positively charged PAH; third row: PEM terminating with a negatively charged PAA layer.

## 4 Discussion

There is a big difference between the roughness of polished and sandblasted surfaces. The roughness (*R*_a_ parameter) of polished samples is 0.08 μm, whereas the sandblasted samples have, as expected, a higher value of roughness, 2.85 μm. The roughness of the terminating PEM does not change substantially (between 63 and 97 nm), and the roughness of PEM does not impact the microbial adhesion. A significant difference between the polished and sandblasted PMMA surfaces was also observed in contact angles. The polished PMMA substrates are hydrophilic with contact angles of (74.9 ± 4.0)°, whereas the sandblasted surfaces are hydrophobic with contact angles of (107.1 ± 4.6)°.

C. albicans adhesion on polished surfaces is higher compared to sandblasted surfaces, as shown in the SEM micrographs shown in Figure 3. The most likely reason for such a difference lies in the presence of aluminum oxide from sandblasting since the presence of antimicrobial aluminum oxide leads to the shape deformation and destruction of C. albicans cells.^35^ Fungal growth on the sandblasted PMMA demonstrates that C. albicans reacts to an unfavorable surface, resulting in the production of pseudohyphae. Sandblasted PMMA has complicated and rough surfaces, and consequently, fewer adhered C. albicans cells are observed. C. albicans virulence is noticeable due to the fungal ability to react to changes in environmental conditions by producing hyphae structures. External stimuli such as pH, serum, CO_2_, and others cause the change from bud-like to filamentous chains of pseudohyphal cells.^36–40^ The development of pseudohypha is related to the increased C. albicans virulence, the production of adhesion proteins that cause expressed attachment to human cells, and biofilm formation, enzymes that facilitate invasive growth and counteract the human immune system defense.^41–44^

The ζ-potential of the terminating layer is crucial for microbial adhesion. The streaming potential measurements in the neutral pH range reveal that the uncoated polished PMMA sample has a ζ-potential of −56.3 mV. PAH-terminating multilayer has a positive ζ-potential of 51.7 mV, whereas the PAA-terminating multilayer has a ζ-potential of −22.2 mV. This is the expected behavior for the PMMA–PAH/PAA system as PMMA is highly negatively charged (ref 100), PAH is positively charged, while PAA has a lower negative charge absolute value. This difference in charge values is expected as PAA is a weak polyelectrolyte and carries a certain amount of uncharged −COOH groups which do not contribute to the ζ-potential value at the measured pH value. After the PEM buildup on the polished PMMA surface, the most pronounced adhesion of C. albicans was observed on positively charged surfaces with a terminating PAH layer. A much lower amount of adhered C. albicans was noticed on the PEMs terminating with a negatively charged PAA layer (see Table 3 and Figure 4). The main reason is the electrostatic repulsion between the negatively charged top layer and the negatively charged fungi, as C. albicans surface charge is also negative.^45^ Similar results were previously observed by Kovačević et al.^15^ showing that in the case of positively charged polyelectrolyte multilayers, the adhesion of negatively charged bacteria is more pronounced than in the case of negatively charged polyelectrolyte layers. Similarly, between negatively charged coatings and negatively charged fungal cells, repulsive forces are expected.^15,46^ This confirms that PAA-coated PMMA showed efficient antifungal properties.

Furthermore, the addition of sucrose increases the adhesion of C. albicans surfaces coated with PAH- and PAA-terminating layers. Sucrose utilization by yeast cells promotes the production of extracellular polysaccharides (EPS) and facilitates the initial stages of biofilm formation. In contrast, hyphal cells primarily depend on their filamentous growth to penetrate and stabilize the biofilm structure, rather than on additional EPS derived from sucrose.^47,48^

In addition, the accelerated cell division after the addition of sucrose can cause enhanced cell attachment to surface which results in faster biofilm formation.^49,50^

Although the choice of coating is important for the initial adhesion of C. albicans, this research indicates that food rich in sucrose and poor maintenance of oral hygiene could help C. albicans cells overcome less favorable coating characteristics and cause biofilm formation. Overall, sucrose intake should be minimized by applying dietary modifications, especially in denture wearers, to mitigate the risks of C. albicans adhesion and biofilm formation. Notably, education of denture wearers on the influence of dietary regimes on oral health should be one of the strategies for preventive care. PEMs terminating with a negatively charged last depositing layer demonstrate potential for antifungal application. However, in the future, different polyelectrolyte combinations to build up a protective coating should be considered.

## 5 Conclusions

The effect of polyelectrolyte multilayer coatings on the properties of PMMA-based dental material was examined with the following main conclusions:

1. PMMA surfaces coated with terminating negatively charged poly­(acrylic) acid layers adhere less C. albicans than surfaces coated with terminating positively charged poly­(allylamine) hydrochloride layer. The lowest amount of adhered C. albicans was obtained on PMMA surfaces coated with a PAA-terminating layer and demonstrated an improved antifungal effect on PMMA surfaces.
2. Sucrose addition increases the bacterial adhesion extent of C. albicans to both types of coated surfaces. With sucrose, the lowest amount of adhered C. albicans was obtained on uncoated PMMA surfaces which means that coating with PEMs (at least in the case of PAH/PAA multilayers) in the presence of sucrose does not improve the antifungal effect of PMMA surfaces.
